# Development of an Analytical Method for Simultaneous Determination of the Modified Forms of 4,15-Diacetoxyscirpenol and their Occurrence in Japanese Retail Food

**DOI:** 10.3390/toxins10050178

**Published:** 2018-04-26

**Authors:** Tomoya Yoshinari, Nanami Takeda, Maiko Watanabe, Yoshiko Sugita-Konishi

**Affiliations:** 1 Division of Microbiology, National Institute of Health Sciences, 3-25-26 Tonomachi, Kawasaki-ku, Kawasaki-shi, Kanagawa 210-9501, Japan; mwatanabe@nihs.go.jp; 2 Department of Food and Life Sciences, Azabu University, 1-17-71 Fuchinobe, Chuo-ku, Sagamihara, Kanagawa 252-5201, Japan; me1703@azabu-u.ac.jp (N.T.); y-konishi@azabu-u.ac.jp (Y.S.-K.)

**Keywords:** 4,15-diacetoxyscirpenol, modified mycotoxin, occurrence, *Fusarium*

## Abstract

4,15-Diacetoxyscirpenol (4,15-DAS) is a type A trichothecene mycotoxin produced by *Fusarium* species. Four modified forms of 4,15-DAS including 7-hydroxydiacetoxyscirpenol, 7,8-dihydroxydiacetoxyscirpenol, 4β,8α,15-triacetoxy-3α,7α-dihydroxy-12,13-epoxytrichothec-9-ene and 4,15-diacetylnivalenol were purified from cultures of *F. equiseti*. An analytical method using a multifunctional column has been developed for the simultaneous determination of 4,15-DAS, its four modified forms, T-2 toxin, HT-2 toxin and neosolaniol in cereals. The performance of the current method was evaluated, and a total of 248 samples of five different commodities were analyzed for over two years by this method. 4,15-DAS was detected in Job’s tears products, corn flour and azuki bean, but it was not found in wheat flour or rye flour. The four modified forms of 4,15-DAS were detected in samples of Job’s tears products, contaminated by 4,15-DAS. This is the first report on quantification of the modified forms of 4,15-DAS in cereals.

## 1. Introduction

**Figure 1:**
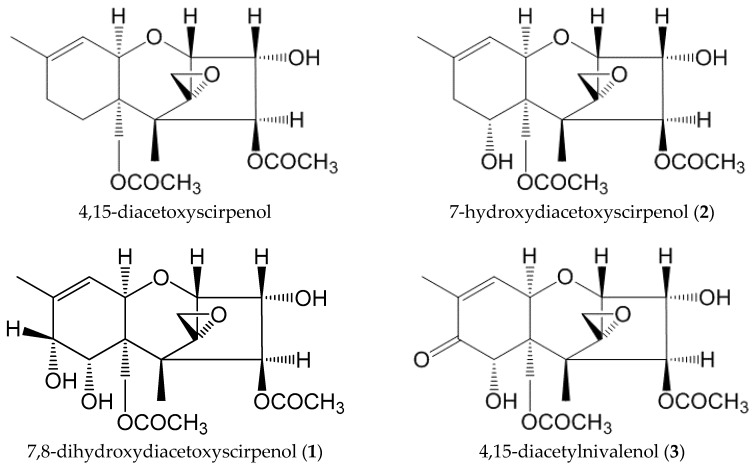

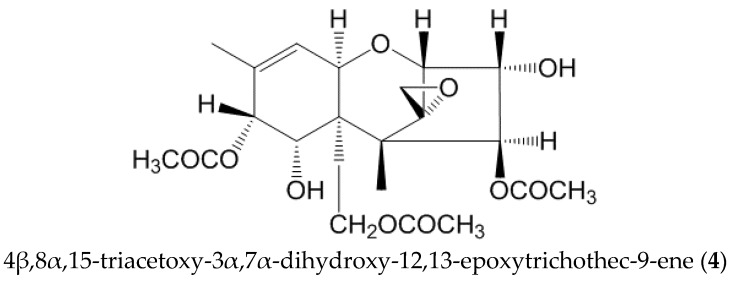
Structures of 4,15-DAS and its modified forms.

Trichothecene mycotoxins are a group of more than 80 compounds mainly produced by the *Fusarium* species. They are classified into four types (A-D) based on their structures [1]. The occurrence of trichothecene mycotoxins in agricultural products have been extensively researched. Our group performed a survey of the contamination of cereals and processed cereal-based foods by deoxynivalenol (DON), a type B trichothecene, and two type A trichothecene mycotoxins, T-2 toxin and HT-2 toxin, between 2010 and 2012 [2]. These trichothecene mycotoxins were detected in a variety of foods including wheat, barley, Job’s tears products, beer, corn flour and azuki bean. 4,15-Diacetoxyscirpenol (4,15-DAS, Figure 1) is a type A trichothecene mycotoxin produced by some *Fusarium* species including *F. equiseti*, *F. poae* and *F. sporotrichioides* [3,4]. As with other trichothecene mycotoxins, acute oral exposure of 4,15-DAS causes diarrhea, lethargy and vomiting in animal models [5,6]. Analytical methods for the simultaneous determination of several trichothecene mycotoxins including 4,15-DAS in foods have been developed. In a survey performed in the UK, the occurrence of trichothecene mycotoxins in wheat and oats was monitored, revealing that DON, nivalenol (NIV), T-2 toxin and HT-2 toxins were detected in samples, while 4,15-DAS was not detected in any samples [7]. In the Canadian cereal grain samples including wheat, barley and corn, 4,15-DAS was not detected in any samples, although DON was frequently found [8]. Unlike DON, NIV, T-2 toxin and HT-2 toxin, information about the contamination of foods by 4,15-DAS is limited. In 2016, the Joint FAO/WHO Expert Committee on Food Additives (JECFA) conducted the first toxicological evaluation of 4,15-DAS [9]. In that meeting, it was decided that 4,15-DAS should be included in the group of provisional tolerable daily intake, which includes T-2 toxin and HT-2 toxin (0.06 μg/kg bw). The committee noted that the occurrence data for 4,15-DAS was insufficient at the time and recommended the quantification of 4,15-DAS in all possible commodities with appropriate sensitivity. 

Recently, a new group of mycotoxin derivatives has been detected in naturally contaminated cereals. They have been termed “modified mycotoxins”, and are mainly produced by a thermal reaction, microbial transformation or plant metabolism [10,11]. Among modified mycotoxins, modified forms of DON including as 3-acetyldeoxynivalenol (3ADON), 15-acetyldeoxynivalenol (15ADON) and deoxynivalenol-3-*O*-β-d-glucopyranoside (D3G) have been well studied. Some analytical methods for the simultaneous determination of DON and its modified forms have been developed and their occurrence in food commodities have been reported [12,13,14]. Recent studies showed that these modified forms of DON were metabolized into DON in vivo, which may exert the same toxic effects as DON [15,16]. In 2017, the European Food Safety Agency (EFSA) evaluated the risk of DON and its modified forms and set a group-TDI for the sum of DON, 3ADON, 15ADON and D3G [17]. In this way, modified mycotoxins were considered as a potential risk to humans.

Some modified forms of 4,15-DAS have been reported. From the culture filtrate of a 4,15-DAS-producing fungus, acetylated or hydroxylated derivatives of 4,15-DAS were purified and identified [18,19]. In the corn powder reference material, glucosides of 4,15-DAS were detected by LC-Orbitrap MS analysis [20]. However, there is little information about the occurrence of modified forms of 4,15-DAS in food. Therefore, it is important to survey the contamination levels of not only 4,15-DAS but also its modified forms to perform a more accurate risk assessment of 4,15-DAS.

In this study, four modified forms of 4,15-DAS were purified from the culture filtrate of a *F. equiseti* strain. Next, an analytical method for the determination of 4,15-DAS and its modified forms were developed, and their occurrence was verified in Japanese retail foods.

## 2. Results

### 2.1. Screening of Fusarium Strains Producing Modified Forms of 4,15-DAS

**Figure 2:**
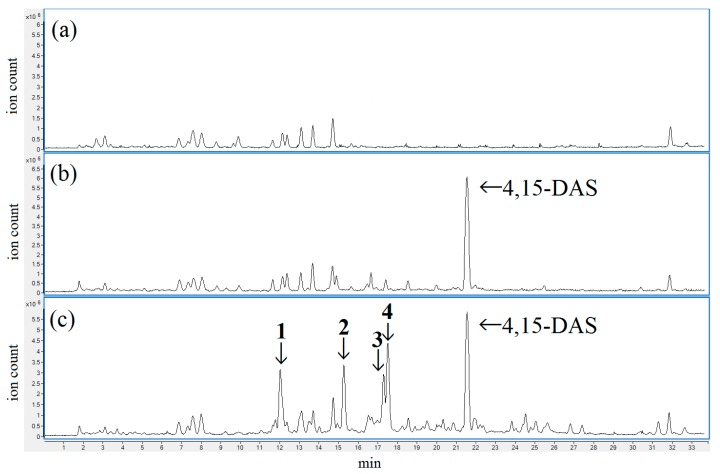
Extracts from the culture broth of Fusarium sp. were subjected to Q-TOF LC/MS. Total ion chromatograms of liquid medium with no fungal growth (**a**) and the culture broth of fungi which produced 4,15-DAS (**b**,**c**).

A total of 66 *Fusarium* strains which had been stocked in our laboratory were separately cultured in liquid medium. Ethyl acetate extracts of each culture broth were subjected to Q-TOF LC/MS. The total ion chromatogram of each culture broth extract was compared with that of a liquid medium extract with no fungal growth (Figure 2a), and the peaks observed only in the culture broth extract were analyzed. 4,15-DAS was detected in the culture broth extracts of 11 FIESC strains. A peak of 4,15-DAS was mainly observed in the total ion chromatograms of the culture broth extracts of eight strains. Figure 2b represents a chromatogram of the culture filtrate from a 4,15-DAS producing strain. Some peaks, including of 4,15-DAS and unidentified compounds, were observed in the chromatograms of the three strains. The estimated molecular formulae of the unidentified compounds were C_19_H_26_O_9_ (compound **1**), C_19_H_29_NO_8_ (compound **2**), C_19_H_27_NO_9_ (compound **3**) and C_21_H_31_NO_10_ (compound **4**). Figure 2c represents a chromatogram of the culture filtrate from a strain which produced 4,15-DAS and compound **1**–**4** (NIHS6453 strain). MS/MS fragmentation analysis revealed some common fragments between 4,15-DAS and the four unidentified compounds. Therefore, the four compounds were assumed to be modified forms of 4,15-DAS. The three FIESC strains, which produced these modified forms of 4,15-DAS, were identified as *F. equiseti* by the morphological observation and the nucleotide sequence analysis of the β-tubulin gene. One of the three *F. equiseti* strains, NIHS6453 strain, was used as a producer of the modified forms of 4,15-DAS.

### 2.2. Identification of the Modified Forms of 4,15-DAS

The *F. equiseti* NIHS6453 strain was cultured on polished rice substrate for two weeks. Methanol extracts of the culture were purified by liquid-liquid extraction, a C18 cartridge and reverse-phase HPLC to isolate the modified forms of 4,15-DAS. The Q-TOF LC/MS, ^1^H- and ^13^C-NMR spectra of the purified compounds (Supplemental Table S1) confirmed that compound **1**, **2**, **3** and **4** were 7,8-dihydroxydiacetoxyscirpenol (7,8-diHDAS), 7-hydroxydiacetoxyscirpenol (7-HDAS), 4,15-diacetylnivalenol (4,15-diANIV) and 4β,8α,15-triacetoxy-3α,7α-dihydroxy-12,13-epoxytrichothec-9-ene, respectively, in comparison to the previous literature [18,19,21]. Chemical structures of the modified forms of 4,15-DAS are shown in Figure 1.

### 2.3. Analytical Methods for Determination of 4,15-DAS and its Modified Forms

**Figure 3:**
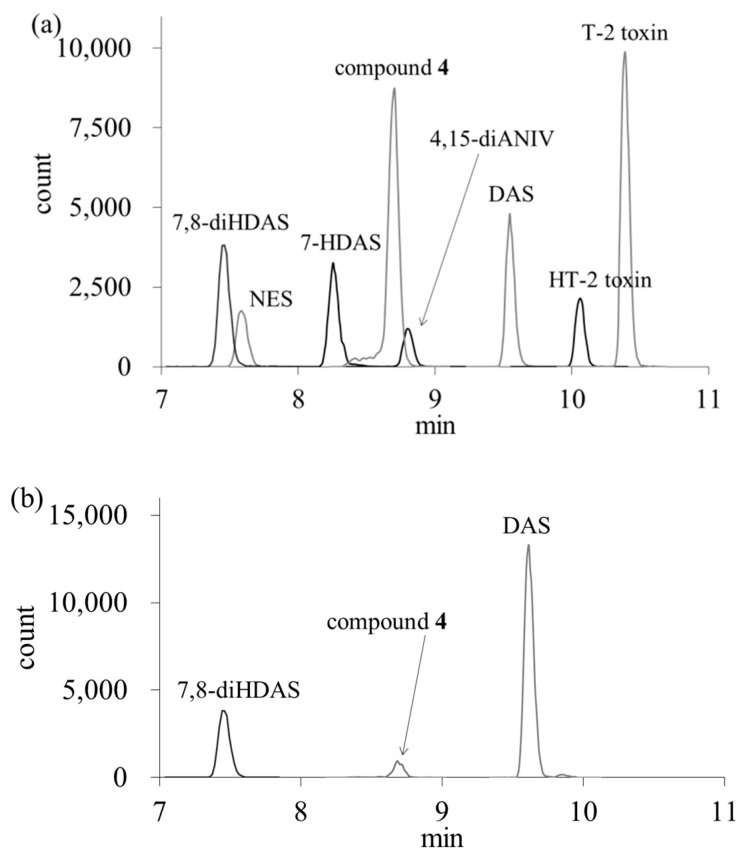
LC-MS/MS chromatograms of a standard solution (**a**) and a contaminated Job’s tears product (**b**). The concentration of each analyte was 5 µg/L. The retention times of 7,8-diHDAS, NES, 7-HDAS, compound **4**, 4,15-diANIV, 4,15-DAS, HT-2 toxin and T-2 toxin were 7.4, 7.6, 8.2, 8.7, 8.8, 9.6, 10.1 and 10.3 min, respectively.

Cleanup of sample extracts was performed with a commercially available multifunctional column, Autoprep MF-T 1500, which is designed for the cleanup of trichothecene mycotoxins. Quantification of the mycotoxins was performed by LC-MS/MS (Supplemental Table S2). In addition to 4,15-DAS and its four modified forms, three type-A trichothecene mycotoxins, T-2 toxin, HT-2 toxin and neosolaniol (NES) were simultaneously quantified. A typical chromatogram of a standard solution of the eight analytes is shown in Figure 3a. To evaluate the performance of the method, it was applied to the analysis with spiked food samples containing eight trichothecene mycotoxins at two levels in five commodities. The recoveries of 4,15-DAS, 7-HDAS, NES, 7,8-diHDAS, 3,4-diANIV, compound **4**, T-2 toxin and HT-2 toxin were in the range of 91–105%, 81–101%, 93–105%, 88–103%, 89–103%, 89–104%, 88–105% and 89–107%, respectively (Table 1). The standard deviations at 5 and 50 μg/kg spiked levels for five commodities ranged from 2 to 14% and from 1 to 9%, respectively. This result shows that the method is applicable for the quantification of eight trichothecene mycotoxins in wheat flour, Job’s tears products, rye flour, corn flour and azuki bean.

### 2.4. Occurrence of 4,15-DAS and its Modified Forms in Cereals

The concentrations of 4,15-DAS, its four modified forms, NES, T-2 toxin and HT-toxin in five different cereals were quantified by an analytical method using a multifunctional column (Table 2). A chromatogram of a contaminated Job’s tears product as an example is shown in Figure 3b. 4,15-DAS was detected in Job’s tears products (63%), corn flour (15%) and azuki bean (9%). The mean concentration of 4,15-DAS in Job’s tears products was 13 µg/kg, and this value was higher than the mean concentration of 4,15-DAS in corn flour (0.07 µg/kg) and azuki bean (0.02 µg/kg). No corn flour or azuki bean samples contained more than 1 µg/kg of 4,15-DAS, while more than 10 µg/kg of 4,15-DAS was detected in 35% of Job’s tears products samples, with the maximum concentration of 70 µg/kg. The four modified forms of 4,15-DAS were detected in Job’s tears products, and the positive rate of 7,8-diHDAS (63%) was the highest among the four modified forms. The mean concentration of 7,8-diHDAS (2 µg/kg) and 4,15-diANIV (2 µg/kg) was higher than that of 7-HDAS (0.1 µg/kg) and compound **4** (0.5 µg/kg). Contamination levels of the four modified forms were lower than that of 4,15-DAS. 7,8-diHDAS was detected in corn flour samples and compound **4** was detected in both corn flour and azuki bean samples, but their mean and maximum concentrations were low compared to Job’s tears products. NES was detected in only 13% of the Job’s tears products samples. T-2 toxin and HT-2 toxin were detected in the five different cereals. The mean concentration of T-2 toxin was the highest in Job’s tears products (2 µg/kg), and the maximum contamination level was detected in a sample of the Job’s tears product (28 µg/kg). The mean HT-2 toxin concentrations of Job’s tears products and rye flour were both 2 µg/kg, and this value was the highest among the five different cereals. The maximum contamination level was present in the rye flour sample (18 µg/kg). In Table 3, the positive rates and the concentrations of type A trichothecene mycotoxins in Job’s tears products are shown separately for each production area. Among the 46 samples of Job’s tears products, 28 samples were domestic, 13 were from Southeast Asia including Thailand and Laos, and the others were unknown. In the Japanese samples, the mycotoxins, which were detected in more than half of the samples, were 4,15-diANIV (68%), T-2 toxin (86%) and HT-2 toxin (68%). In Job’s tears products from Southeast Asia, 4,15-DAS, 7,8-diHDAS and compound **4** were detected in all samples, while T-2 toxin and HT-2 toxin were not found in any samples.

## 3. Discussion

In this study, T-2 toxin and HT-2 toxin were detected in the five different cereals, while 4,15-DAS was found in three cereals including Job’s tears products, corn flour and azuki bean. In corn flour and azuki bean, the contamination level of 4,15-DAS was much lower than that of T-2 toxin and HT-2 toxin. In contrast, 4,15-DAS was frequently detected in Job’s tear products, and its mean and maximum concentrations were greater than those of T-2 toxin and HT-2 toxin. Interestingly, the contamination pattern of type A trichothecene mycotoxins in Japanese Job’s tears products was different to that of Southeast Asian samples. In the Japanese samples, T-2 toxin and HT-2 toxin were mainly detected, and the contamination level of 4,15-DAS was less than that of T-2 toxin and HT-2 toxin. Conversely, 4,15-DAS was found at a large frequency in the Southeast Asian samples, but T-2 toxin and HT-2 toxin were not detected at all. The production zone of Job’s tears in Japan belongs to a mild and a humid climate, while Southeast Asian countries are located in a torrid zone. This indicates that climate is one of the factors influencing the pattern of type A trichothecene mycotoxin contamination in cereals, in which 4,15-DAS contamination is more likely to occur in hot and humid areas. The countries of origin of the wheat flour samples in our survey were Australia, Canada, France, Japan and the United States. The wheat production areas of these countries are not located in torrid zones, which may explain why 4,15-DAS was not detected in our wheat flour samples. In other surveys performed in European countries, the contamination level of 4,15-DAS in wheat samples was less than that of T-2 toxin and HT-2 toxin [22], in which our results indicated similar findings. 

The compounds purified from the culture broth of *F. equiesti* were considered to be modified forms of 4,15-DAS because their chemical structures were quite similar to that of 4,15-DAS. They have already been identified as metabolites from *Fusarium* sp. [18,19,21], but their occurrence in foods have not been studied at all. As the result of this survey, four modified forms of 4,15-DAS were detected in Job’ tears products. We found that the modified forms of 4,15-DAS were produced not only in liquid culture media but also in their natural environment. The trichothecene biosynthetic pathway in *Fusarium* species have been well studied, and many trichothecene biosynthetic enzymes were identified [23]. Among them, TRI1 in *Fusarium graminearum* is a P450 oxygenase which catalyzes the oxidation of both C-7 and C-8 of calonectrin, an intermediate of the DON biosynthetic pathway [24]. TRI16 in *Fusarium sporotrichioides* is an acyltransferase which catalyzes the formation of an ester side group at C-8 [25]. These enzymes could non-specifically modify 4,15-DAS, and the modified forms were found in the cereals.

The toxicity of the modified forms of 4,15-DAS has been studied previously. The LD_50_ values of 4,15-DAS, 7-HDAS, 7,8-diHDAS and 4,15-diANIV to mice dosed intraperitoneally were 23.0, 3.5, 6.0 and 9.6 mg/kg, respectively [26,27]. The LD_50_ value of compound **4** in rats dosed intraperitoneally was 1.2 mg/kg [18]. These results show that the modified forms of 4,15-DAS are as toxic as 4,15-DAS. In the samples of Job’s tears products in our survey, the modified forms of 4,15-DAS were found in all of the 4,15-DAS-contaminated samples. A detailed toxicological study is needed to consider a necessity of including the modified forms of 4,15-DAS in the health risk assessment of 4,15-DAS in food.

## 4. Materials and Methods 

### 4.1. Chemicals, Samples, and Strains

Solid crystals of 4,15-DAS, T-2 toxin, HT-2 toxin and NES were purchased from Sigma-Aldrich (St. Louis, MO, USA). Each compound was dissolved in acetonitrile (100 mg/L each) and the stock solution was stored at −30 °C. LC-MS-grade acetonitrile and water, and reagent-grade acetonitrile and ammonium acetate were purchased from Wako Pure Chemicals (Osaka, Japan). The retail food was purchased randomly from local supermarkets and small retail shops in Japan from the spring of 2016 to the winter of 2017. *Fusarium* strains were isolated in 2013 from soybean or azuki bean purchased from retail shops in Japan. These strains were morphologically identified as *Fusarium avenaceum*, *Fusarium oxysporum*, *Fusarium proliferatum*, *Fusarium incarnatum-equiseti* species complex (FIESC) or *Fusarium* sp.

### 4.2. Sample Preparation for Q-TOF LC/MS Analysis

A piece of mycelium from a 10 day-old potato dextrose agar slant culture of a strain was introduced into a 300 mL flask containing 50 mL of a liquid medium (0.05% KCl, 0.05% MgSO_4_, 0.1% K_2_HPO_4_, 0.2% NaNO_3_, 0.25% yeast extract, 0.5% polypeptone and 5% sucrose), and static incubation was performed for 7 d at 27 °C. The culture filtrate (200 μL) was extracted with 200 μL of ethyl acetate and the ethyl acetate solution was evaporated to dryness in a stream of nitrogen at 40 °C. The residue obtained was dissolved in 1 mL of methanol and was subjected to Q-TOF LC-MS analysis.

### 4.3. Q-TOF LC-MS Analysis Conditions

The LC system consisted of an Agilent 1200 series (Agilent Technologies, Palo Alto, CA, USA). The LC conditions were as follows: mobile phase, 10 mmol/L ammonium acetate/acetonitrile; linear gradient of 10–82% acetonitrile for 40 min, linear gradient of 82–10% acetonitrile for 1 min, followed by equilibration at 10% acetonitrile for 20 min before the next injection. The flow rate was 0.2 mL/min, and the column used was a 150 mm × 2.1 mm i.d., 3 μm, InertSustain C18 (GL Sciences Inc., Tokyo, Japan). The column oven was held at 40 °C, and the autosampler tray was maintained at 4 °C. The HPLC system was connected to an Agilent 6530 Q-TOF mass spectrometer. An electrospray ionization (ESI) interface with Agilent Jet Stream Technology was used in the positive mode. The instrument was calibrated in the high resolution mode (4 GHz) with a standard mass range (*m*/*z* < 3200). Reference masses at *m*/*z* 121.0508 and 922.0098 were continually introduced with the LC stream for accurate mass calibration. The drying gas (nitrogen) temperature was set at 325 °C, drying gas flow at 10 L/min, nebulizer pressure at 30 psi, and capillary voltage (Vcap) at 3500 V. A centroid data within the mass range *m*/*z* 100−1000 was acquired at a 1 spectrum/s rate with a Mass Hunter workstation (Agilent Technologies, Palo Alto, CA, USA). Peak identification was performed using Qualitative Analysis software version B.04.00 (Agilent Technologies, Palo Alto, CA, USA). The criteria for peak identifications were as follows; height: >10,000 counts, peak spacing tolerance: 0.0025 *m*/*z* plus 7.0 ppm.

### 4.4. Purification of the Modified Forms of 4,15-DAS

The *F. equiseti* NIHS6453 strain was cultured in the above-mentioned liquid medium for 48 h at 27 °C while shaking at 100 rpm. Polished rice (180 g) used as a substrate was soaked in water for 3 h and autoclaved. The rice was equally divided into six 300 mL Erlenmeyer flasks, and water (12 mL) was added to each flask. After autoclaving, 5 mL of the strain’s culture broth was added to each flask. The culture was incubated at 27 °C in the dark, and harvested after two weeks. The modified forms of 4,15-DAS were extracted by homogenizing the culture with 720 mL of acetonitrile: water (85:15). The filtrate was collected by vacuum filtration and was dried by removing the solvent in a rotary evaporator. The residue was suspended in 100 mL of water, and the suspension was extracted twice with the 100 mL of ethyl acetate. The ethyl acetate layer was evaporated to dryness and the residue was suspended in 5 mL of methanol. After centrifugation (3000 g, 10 min), 0.5 mL of the supernatant was subjected to an HF Mega BE-C18 (5 g) cartridge (Agilent Technologies, Palo Alto, CA, USA), which was preequilibrated with 20 mL of water. The column was washed with 20 mL of water and 20 mL of 10% acetonitrile in water. The modified forms of 4,15-DAS were eluted with 20 mL of 20% acetonitrile in water (fraction A) and 20 mL of 50% acetonitrile in water (fraction B). This process was repeated until all of the supernatants were treated. Fractions A and B were separately evaporated to dryness. The residue of fraction A was subjected to an LC-20A series HPLC system (Shimadzu Corp., Kyoto, Japan). The column used was a 250 mm × 10 mm i.d., 5 μm, Inertsil ODS-3 (GL Sciences Inc., Tokyo, Japan). Isocratic elution with 15% acetonitrile in water, at a flow rate of 4.0 mL/min with detection at 200 nm was used to obtain 7,8-dihydroxydiacetoxyscirpenol (20.0 min retention time, 21 mg yield, HRESI-TOF/MS *m*/*z* 416.1917 [M + NH_4_]^+^, calcd. for C_19_H_26_O_9_, 398.1577). The residue of fraction B was subjected to reverse-phase HPLC, equipped with the same column. Linear gradient elution of 25–50% acetonitrile in water from 0–15 min, and isocratic elution of 50% acetonitrile in water from 15–30 min at a flow rate of 4.0 mL/min with detection at 200 nm, were used to obtain 7-hydroxydiacetoxyscirpenol (12.6 min retention time, 45 mg yield, HRESI-TOF/MS *m*/*z* 400.1970 [M + NH4]+, calcd. for C19H26O8, 382.1628), fraction C (15.4–17.8 min) and 4,15-diacetylnivalenol (19.8 min retention time, 19 mg yield, HRESI-TOF/MS *m*/*z* 414.1762 [M + NH_4_]^+^, calcd. for C_19_H_24_O_9_, 396.1419). Fraction C was purified by the reverse-phase of HPLC equipped with the same column. Linear gradient elution of 10–40% acetonitrile in water from 0–45 min at a flow rate of 4.0 mL/min with detection at 220 nm, was used to obtain 4β,8α,15-triacetoxy-3α,7α-dihydroxy-12,13-epoxytrichothec-9-ene (32.0 min retention time, 38 mg yield, HRESI-TOF/MS *m*/*z* 458.2027 [M + NH_4_]^+^, calcd. for C_21_H_28_O_10_, 440.1683). ^1^H-NMR and ^13^C-NMR spectra of purified compounds were obtained using a Bruker DRX-500 spectrometer. These data are shown in Supplementary Table S1. Each compound was dissolved in acetonitrile (10 g/L each), and the stock solution was stored at −30 °C.

### 4.5. Analytical Method for Simultaneous Determination of the Modified Forms of 4,15-DAS

Twenty five grams of a sample was extracted with 100 mL of acetonitrile in water (85:15, *v*/*v*). The extraction was performed on a horizontal shaker for 30 min at 180 rpm, then the extract was filtered through a filter paper (Toyo Roshi Kaisha, Tokyo, Japan). A portion (10 mL) of the filtered extract was transferred into a multifunctional column, Autoprep MF-T 1500 (Showa Denko K. K., Tokyo, Japan), without pre-conditioning. For wheat flour, Job’s tears products, rye flour and corn flour, the first 3 mL of the eluate was discarded, and the next 2.4 mL was collected. Two milliliters of the eluate was transferred to a test tube. For azuki bean, the first 5 mL of the eluate was collected, and 2 mL of the eluate was transferred to a test tube. The collected eluate in a test tube was dried under nitrogen at approximately 40 °C. The residue was dissolved in 500 μL of acetonitrile in water (10:90, *v*/*v*). After centrifugation (10,000× *g*, 5 min), the sample solution was subjected to LC-MS/MS analysis. 

### 4.6. LC-MS/MS Analysis Conditions

LC-MS/MS analyses were performed with a 3200 Q TRAP LC-MS/MS system (AB Sciex, Foster City, CA, USA) equipped with an ESI source and an LC-20A series high-performance LC system (Shimadzu Corp., Kyoto, Japan). The column used was a 150 mm × 2.1 mm i.d., 3 μm, InertSustain C18 (GL Sciences Inc., Tokyo, Japan). Chromatographic separation was achieved at 40 °C, using gradient elution of 20–90% acetonitrile in water containing 2 mmol/L ammonium acetate from 0–12 min at a flow rate of 0.2 mL/min. The total running time was 17 min, including the 5 min of equilibration. The injection volume was 10 μL. The ESI source was operated at 400 °C in the positive ionization mode. Other MS parameters were as follows; curtain gas: 10 psi, ion spray voltage: 5000 V, nebulizer gas (GS1): 70 psi, turbo heater gas (GS2): 50 psi, collision-activated dissociation gas: 3 (arbitrary units), scheduled multiple reaction monitoring (MRM): on, MRM detection window: 40 s, target scan time: 3 s, pause between mass ranges: 5 ms. The MRM transitions are shown in Supplementary Table S2.

### 4.7. Performance Evaluation of the Analytical Method

Portions of the stock solutions of the eight trichothecene mycotoxins were mixed and diluted with acetonitrile to make two mixed spiking solutions (500 µg/L and 5 mg/L each). Recovery experiments were performed by adding 250 µL of the mixed spiking solutions to 25 g of a non-contaminated sample. The final concentrations were 5 and 50 µg/kg for each analyte. After incubating for 1 h at room temperature, eight compounds in the spiked samples were quantified by the method mentioned above.

### 4.8. Calibration Curve

The calibration curve was used to calculate the limits of detection (LODs), limits of quantification (LOQs) and the concentrations of mycotoxins in the sample solution. The stock solutions of the eight trichothecene mycotoxins were mixed and diluted by acetonitrile in water (10:90, *v*/*v*), and the seven standard solutions with different concentrations (0.1, 0.3, 1.0, 3.0, 10, 30, 100 ng/mL) were prepared. Detected concentrations were calculated on a peak-area basis using AB SCIEX Analyst version 1.6.2 software. LODs and LOQs were calculated on the basis of signal-to-noise ratios of 3:1 and 10:1, respectively.

## Figures and Tables

**Table 1:** Spiking concentrations and recovery of toxins for validation of the methods.

Commodity	Conc. in Spiked Sample (μg/kg)	Recovery (%) ^a^																							
		4,15-DAS			7-HDAS			NES			7,8-diHDAS			4,15-diANIV			compound 4			T-2 Toxin			HT-2 Toxin		
Wheat flour	5	98	±	2	94	±	9	101	±	6	100	±	5	103	±	5	99	±	5	91	±	4	97	±	4
	50	100	±	6	97	±	8	98	±	7	99	±	8	93	±	8	96	±	7	92	±	6	97	±	5
Job’s tears products	5	94	±	5	94	±	6	93	±	6	96	±	4	91	±	8	91	±	5	101	±	3	98	±	7
	50	93	±	5	99	±	3	98	±	6	99	±	7	96	±	6	97	±	5	94	±	6	95	±	7
Rye flour	5	97	±	3	81	±	6	100	±	4	88	±	2	94	±	3	89	±	3	96	±	2	103	±	4
	50	100	±	2	89	±	6	100	±	3	93	±	2	89	±	3	92	±	4	98	±	1	100	±	1
Corn flour	5	105	±	4	101	±	8	105	±	4	102	±	4	102	±	5	99	±	5	105	±	2	107	±	3
	50	95	±	3	97	±	2	101	±	4	100	±	2	95	±	1	91	±	3	95	±	3	103	±	3
Azuki bean	5	101	±	5	92	±	8	101	±	13	103	±	14	99	±	5	104	±	7	91	±	4	99	±	4
	50	91	±	3	88	±	6	93	±	9	97	±	9	90	±	3	99	±	3	88	±	2	89	±	1

^a^ Values are expressed as mean ± standard deviation (n = 6).

**Table 2:** Occurrence of 4,15-DAS, its modified forms, NES, T-2 toxin and HT-2 toxin in Japanese retail food.

Analyte	LOD/LOQ (µg/kg)		Commodity				
			Wheat Flour	Job’s Tears Product	Rye Flour	Corn Flour	Azuki Bean
		No. of Sample	101	46	41	27	33
4,15-DAS		Positive rate (%) ^a^	0	63	0	15	9
	0.1/0.2	Mean (µg/kg)	-	13	-	0.07	0.02
		Maximum (µg/kg)	-	70	-	0.8	0.3
7-HDAS		Positive rate (%)	0	13	0	0	0
	0.2/0.5	Mean (µg/kg)	-	0.1	-	-	-
		Maximum (µg/kg)	-	2	-	-	-
NES		Positive rate (%)	0	13	0	0	0
	0.2/0.5	Mean (µg/kg)	-	0.2	-	-	0
		Maximum (µg/kg)	-	3	-	-	-
7,8-diHDAS		Positive rate (%)	0	63	0	7	0
	0.1/0.2	Mean (µg/kg)	-	2	-	0.07	-
		Maximum (µg/kg)	-	10	-	1	-
4,15-diANIV		Positive rate (%)	0	50	0	0	0
	0.2/0.5	Mean (µg/kg)	-	2	-	-	-
		Maximum (µg/kg)	-	16	-	-	-
compound **4**		Positive rate (%)	0	41	0	7	6
	0.1/0.2	Mean (µg/kg)	-	0.5	-	0.02	0.03
		Maximum (µg/kg)	-	4	-	0.4	0.6
T-2 toxin		Positive rate (%)	9	41	56	22	36
	0.1/0.2	Mean (µg/kg)	0.04	2	0.5	0.1	0.9
		Maximum (µg/kg)	1	28	4	1	8
HT-2 toxin		Positive rate (%)	26	41	76	15	55
	0.1/0.4	Mean (µg/kg)	0.4	2	2	0.1	1
		Maximum (µg/kg)	4	14	18	1	6

^a^ Percentage > LOQ.

**Table 3:** Occurrence of type A trichothecene mycotoxins in Job’s tears products for each production area.

Analyte		Area		
		Japan	South-East Asia	Unknown
	No. of Sample	28	13	5
4,15-DAS	Positive rate (%) ^a^	39	100	100
	Mean (µg/kg)	0.9	25	48
7-HDAS	Positive rate (%)	4	23	40
	Mean (µg/kg)	0.06	0.2	0.4
NES	Positive rate (%)	21	0	0
	Mean (µg/kg)	0.3	-	-
7,8-diHDAS	Positive rate (%)	39	100	100
	Mean (µg/kg)	0.5	4	5
4,15-diANIV	Positive rate (%)	68	15	40
	Mean (µg/kg)	2	0.1	0.9
compound 4	Positive rate (%)	4	100	100
	Mean (µg/kg)	0.03	1	1
T-2 toxin	Positive rate (%)	68	0	0
	Mean (µg/kg)	4	-	-
HT-2 toxin	Positive rate (%)	68	0	0
	Mean (µg/kg)	3	-	-

^a^ Percentage > LOQ.

## Supplementary Materials

The following are available online at http://www.mdpi.com/2072-6651/10/5/178/s1, Table S1: NMR spectroscopic data for the modified forms of 4,15-DAS, Table S2: LC-MS/MS parameters for the method developed in the present study.
