# Supplementary material for: Development of an Analytical Method for Simultaneous Determination of the Modified Forms of 4,15-Diacetoxyscirpenol and their Occurrence in Japanese Retail Food
Source: Toxins (Basel). 2018 Apr 26;10(5):178. doi: 10.3390/toxins10050178 (PMC5983234; doi:10.3390/toxins10050178)
Supplement: Supplementary file 1 [file toxins-10-00178-s001.pdf]

Supplementary Materials: Development of an Analytical Method for Simultaneous Determination of the Modified Forms of 4,15-Diacetoxyscirpenol and their Occurrence in Japanese Retail Food

Tomoya Yoshinari, Nanami Takeda, Maiko Watanabe and Yoshiko Sugita-Konishi

Table S1. NMR spectroscopic data for modified forms of 4,15-DAS.

position	7-HDAS (2)		
	δ_C [a]	δ_H (*J* in Hz) [a]	HMBC
2	81.6	3.54, d (4.7)	4, 5, 11, 12
3	78.2	4.20, m	4
4	84.7	5.85, d (3.2)	3, 5, 6, 12, <u>C</u>OCH$_3$
5	50.9		
6	48.8		
7	68.7	4.58, dd (10.1, 6.3)	5, 6, 11, 15
8	40.4	2.14, dd	6, 7, 9, 10
		2.32, dd (18.3, 6.3)	
9	140.4		
10	120.3	5.47, m	
11	71.6	4.49, d (6.0)	7, 9, 10, 15
12	65.7		
13	47.5	3.03, d (4.4)	2, 12
		3.07, d (4.4)	
14	9.1	1.04, s	4, 5, 6, 12
15	63.5	4.18, d (12.4)	5, 6, 11, 17, <u>C</u>OCH$_3$
		4.57, d (12.4)	
16	22.8	1.73, s	8, 9, 10
CO<u>C</u>H$_3$	21.0	2.10, s	<u>C</u>OCH$_3$
CO<u>C</u>H$_3$	21.2	2.07, s	<u>C</u>OCH$_3$
<u>C</u>OCH$_3$	172.5		
<u>C</u>OCH$_3$	173.0		

[a] 125 MHz (δ_C) and 500 MHz (δ_H) in CD_3OD.

position	7,8-diHDAS (3)	
	δ_C [a]	δ_H (*J* in Hz) [a]
2	79.9	3.56, d (4.4)
3	76.7	4.19, m
4	83.1	5.86, d (2.8)
5	49.2	
6	45.9	
7	69.4/69.6/69.7 [b]	3.94, d (5.1)
8	69.4/69.6/69.7 [b]	4.26, d (5.1)
9	140.3	
10	120.3	5.59, d (5.2)
11	69.4/69.6/69.7 [b]	4.52, d (5.2)
12	63.4/64.2 [c]	

13	48.3	3.03, d (3.7)
		3.11, d (3.7)
14	7.7	1.08, s
15	63.4/64.2 [c]	4.46, d (6.8)
		4.48, d (6.8)
16	19.8	1.86, s
CO<u>C</u>H3	19.2	2.08, s
CO<u>C</u>H3	19.4	2.09, s
<u>C</u>OCH3	170.9	
<u>C</u>OCH3	171.3	

[a] 125 MHz (δ_C) and 500 MHz (δ_H) in CD_3OD, [b] Assignments uncertain, [c] Assignments uncertain.

position	Compound 4	
	δ_C [a]	δ_H (*J* in Hz) [a]
2	79.9	3.57, d (4.9)
3	76.6	4.20, m
4	83.0	5.82, d (3.2)
5	49.3	
6	45.8	
7	68.1/69.4/71.2 [b]	4.67, d (5.5)
8	68.1/69.4/71.2 [b]	5.75, d (5.5)
9	136.1	
10	123.8	5.82, d (5.7)
11	68.1/69.4/71.2 [b]	4.56, d (5.7)
12	62.7/64.0 [c]	
13	46.9	3.03 ,d (4.1)
		3.09, d (4.1)
14	7.6	1.03, s
15	62.7/64.0 [c]	4.40, d (11.8)
		4.51, d (11.8)
16	18.7	1.73, s
CO<u>C</u>H3	19.4	2.08, s
CO<u>C</u>H3	19.5	2.09, s
CO<u>C</u>H3	19.7	2.10, s
<u>C</u>OCH3	170.9	
<u>C</u>OCH3	171.3	
<u>C</u>OCH3	171.4	

[a] 125 MHz (δ_C) and 500 MHz (δ_H) in CD_3OD, [b] Assignments uncertain, [c] Assignments uncertain.

position	4,15-diANIV (5)		
	δ_C [a]	δ_H (*J* in Hz) [a]	HMBC
2	81.7	3.69, d (5.0)	4, 5, 11, 12
3	78.1	4.29, m	4
4	83.7	5.63, d (3.5)	3, 5, 6, 12, <u>C</u>OCH3
5	51.1		
6	53.8		
7	74.8	4.87, s	5, 6, 8, 15
8	200.8		
9	137.5		
10	139.3	6.62, d (5.8)	6, 8, 11, 16
11	70.7	4.76, d (5.8)	7, 9, 10, 15
12	65.7		

13	47.0	3.03, d (4.2)	2, 12
		3.06, d (4.2)	
14	8.2	0.98, s	4, 5, 6, 12
15	63.3	4.15, d (12.4)	5, 6, 11, 17, $\underline{C}OCH_3$
		4.58, d (12.4)	
16	15.5	1.86, s	8, 9, 10
$CO\underline{C}H_3$	20.8	1.93, s	$\underline{C}OCH_3$
$CO\underline{C}H_3$	20.8	2.10, s	$\underline{C}OCH_3$
$\underline{C}OCH_3$	172.2		
$\underline{C}OCH_3$	172.5		

[a] 125 MHz (δ_C) and 500 MHz (δ_H) in CD_3OD.

Table S2. LC-MS/MS parameters for the developed method in the present study.

Analyte	Precursor ion (*m*/*z*)	Product ion (*m*/*z*) [a]	Collision energy (eV)
4,15-diacetoxyscirpenol	384	307	17
		247	19
7-hydroxydiacetoxyscirpenol	400	105	37
		215	21
neosolaniol	400	185	25
		215	21
7,8-dihydroxydiacetoxyscirpenol	416	121	31
		213	25
4,15-diacetylnivalenol	414	247	21
		137	31
4β,8α,15-triacetoxy-3α,7α-dihydroxy-12,13-epoxytrichothec-9-ene	458	121	31
		213	23
T-2 toxin	484	215	25
		185	29
HT-2 toxin	442	215	19
		263	21

[a] top: quantifier ion, bottom: qualifier ion.
